# Supplementary material for: Graphene balls for lithium rechargeable batteries with fast charging and high volumetric energy densities
Source: Nat Commun. 2017 Nov 16;8:1561. doi: 10.1038/s41467-017-01823-7 (PMC5691064; doi:10.1038/s41467-017-01823-7)
Supplement: Supplementary file 1 — Supplementary Information [file 41467_2017_1823_MOESM1_ESM.pdf]

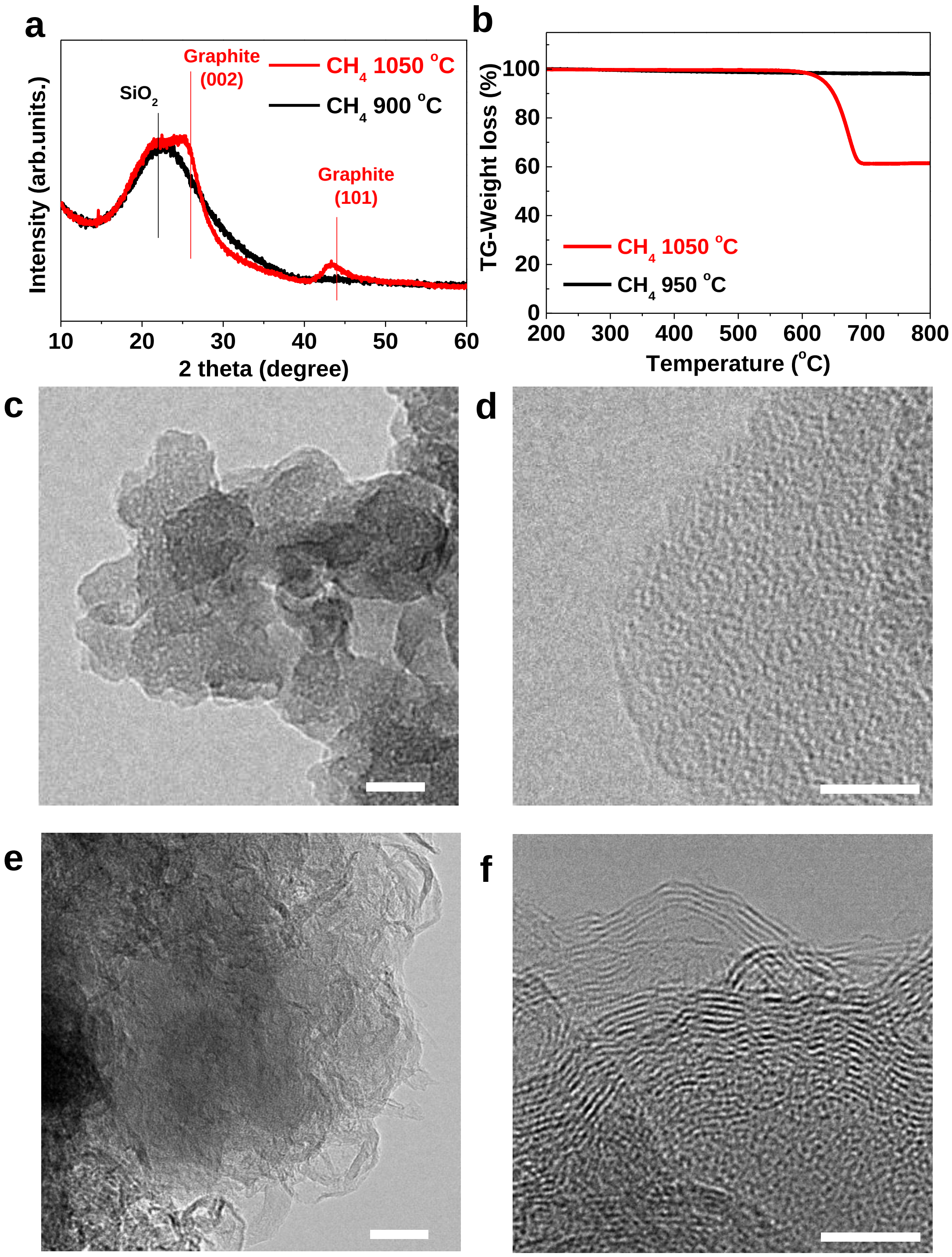

Supplementary Figure 1 | Graphene growth from SiO_2 nanoparticles for 10 min using CH_4 only. (a-b) a, XRD spectra and **b,** TGA profiles for the samples processed at 900 °C and 1050 °C. **(c-f)** TEM images of the samples processed at **(c-d)** 900 °C (*scale bars*, 20 nm and 5 nm, respectively) and **(e-f)** 1050 °C (*scale bars*, 20 nm and 5 nm, respectively).

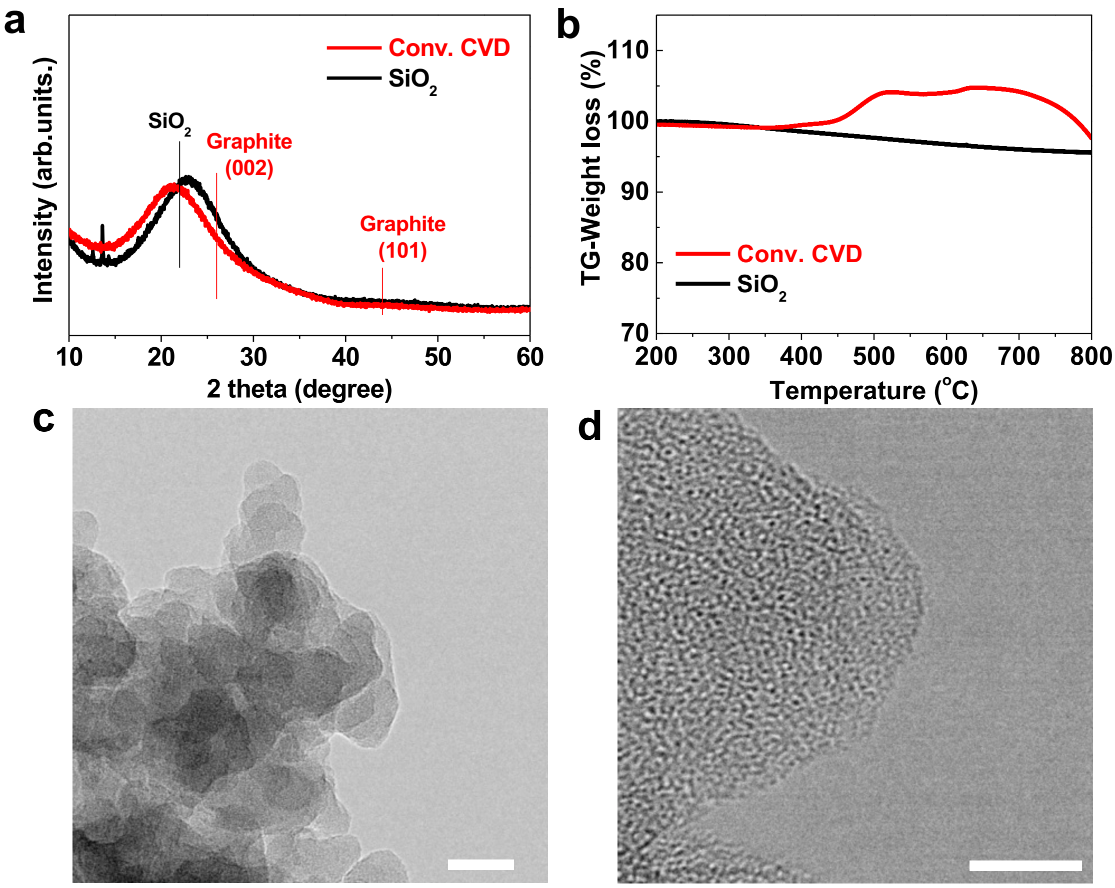

Supplementary Figure 2 | Graphene growth from SiO_2 nanoparticles by a conventional CVD process (H_2 + CH_4, 1000 ^oC) for 10 min. a, XRD spectra. **b,** TGA profiles. **c,** SEM image (*scale bar*, 20 nm). **d,** TEM image (*scale bar*, 5 nm). The series of analyses indicate negligible graphene growth.

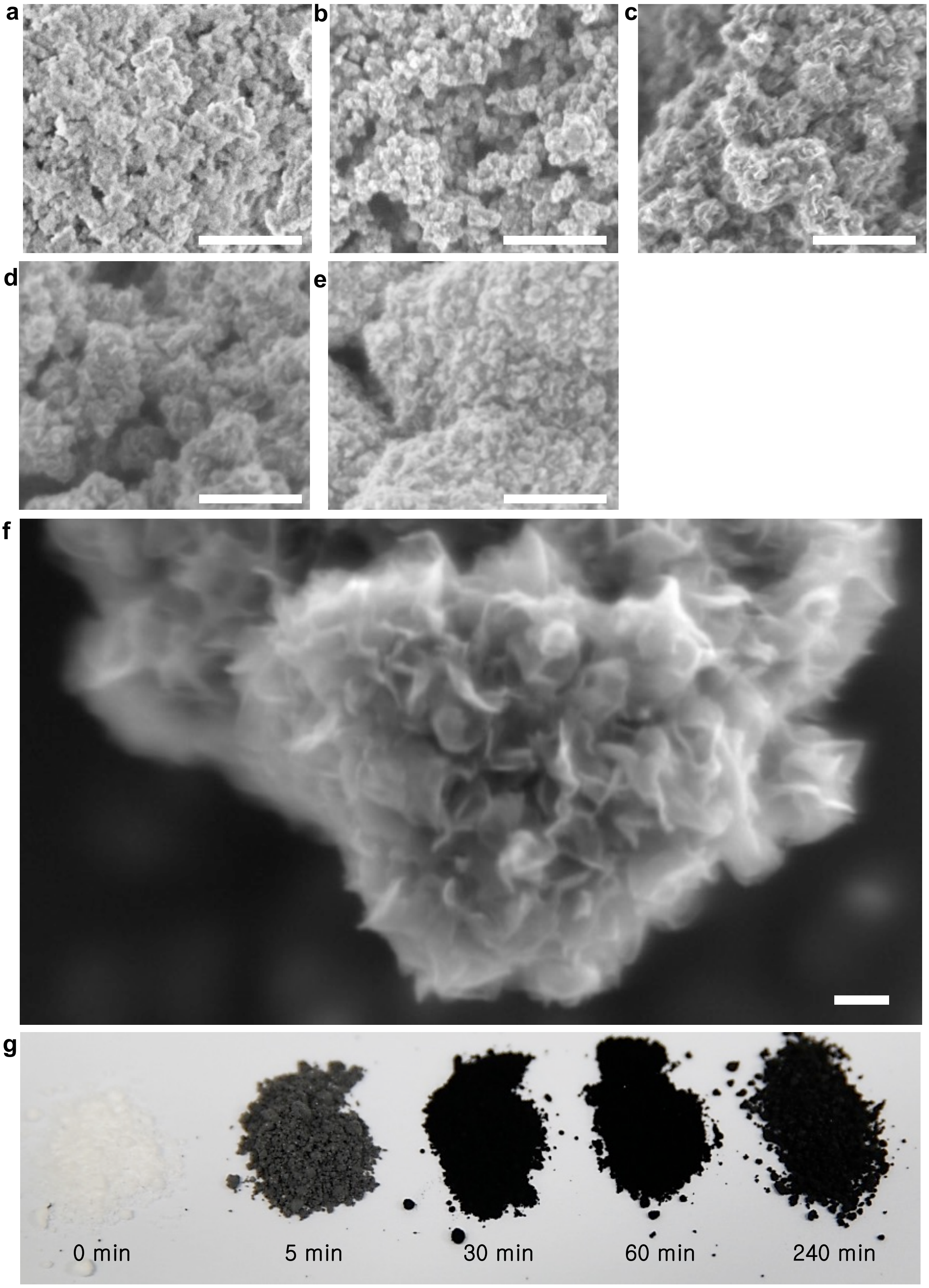

Supplementary Figure 3 | Morphology and color of GB at different CVD growth time. **(a-e)** SEM images of GB after **a,** 0 min, **b,** 5 min, **c,** 30 min, **d,** 60 min, and **e,** 240 min (*scale bars*, 500 nm). **f,** Higher magnification SEM image of **c** (*scale bar,* 50 nm). **g,** Photographs of GB powder at different CVD growth time.

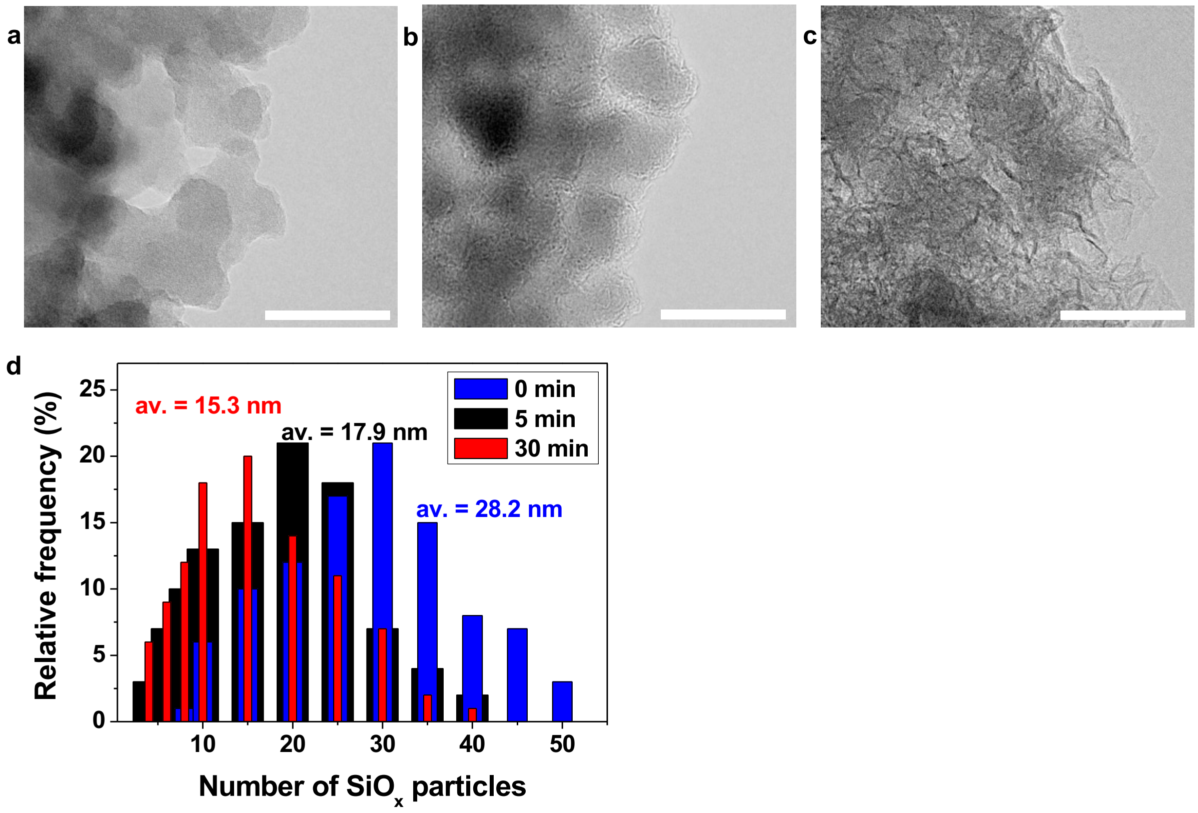

Supplementary Figure 4 | Graphene growth from SiO_2 nanoparticles. (a-c) TEM images after different growth time. **a,** 0 min, **b,** 5 min, and **c,** 30 min min (*scale bars*, 50 nm). **d**, Size change of SiO_2 particles during the course of the CVD process.

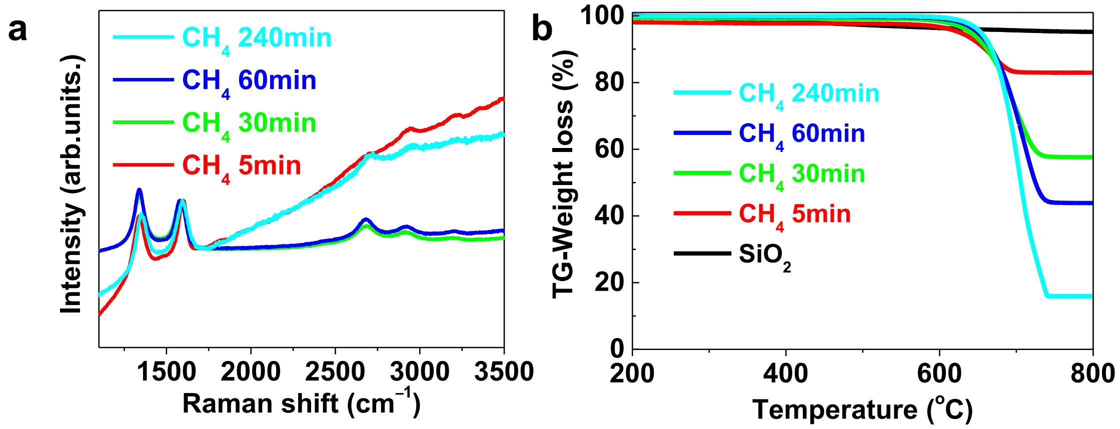

Supplementary Figure 5 | Characterization of GB during the course of graphene growth. **a,** Raman spectra. **b,** TGA profiles.

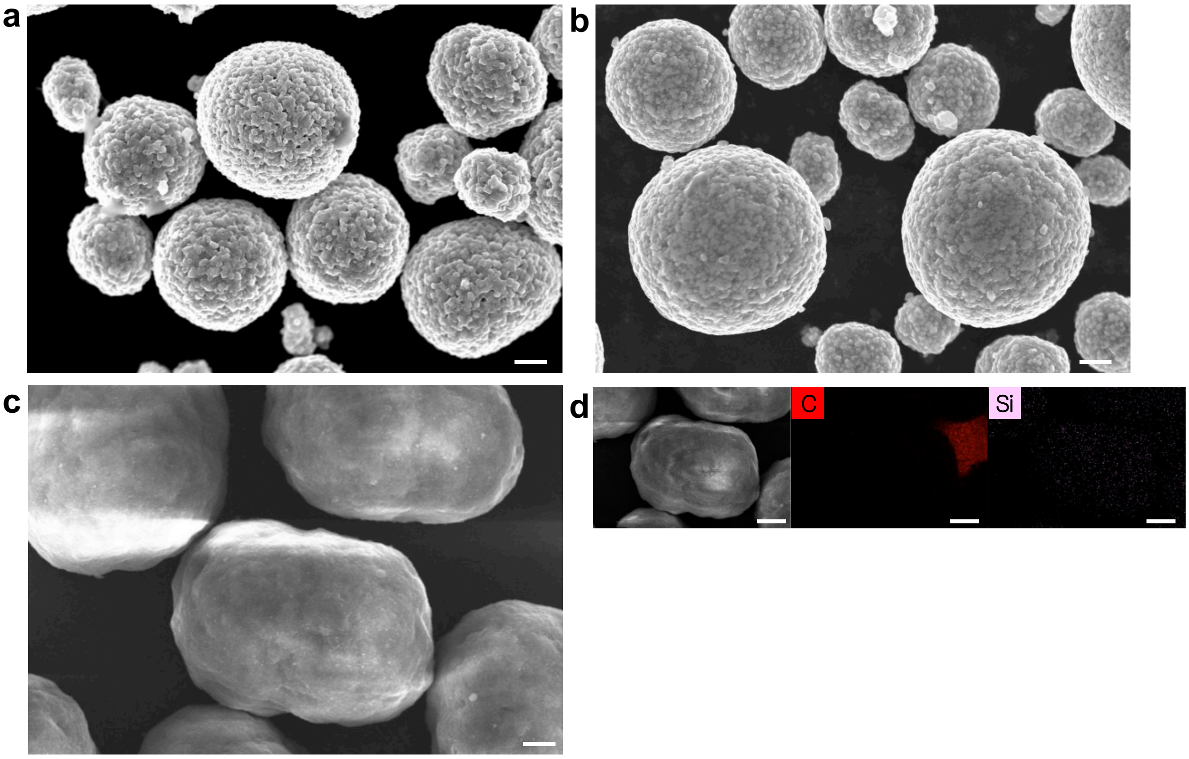

Supplementary Figure 6 | Morphology characterization before and after surface coating. (a-b) SEM images (*scale bars*, 2 µm) **a,** before and **b,** after GB coating. **c,** SEM image (*scale bar*, 1 µm) after the coating with a physical mixture of 0.5 wt% SiO_2 particles and 0.5 wt% graphene. **d,** EDX mapping of the sample shown in c with respect to carbon and silicon (*scale bars*, 2 µm).

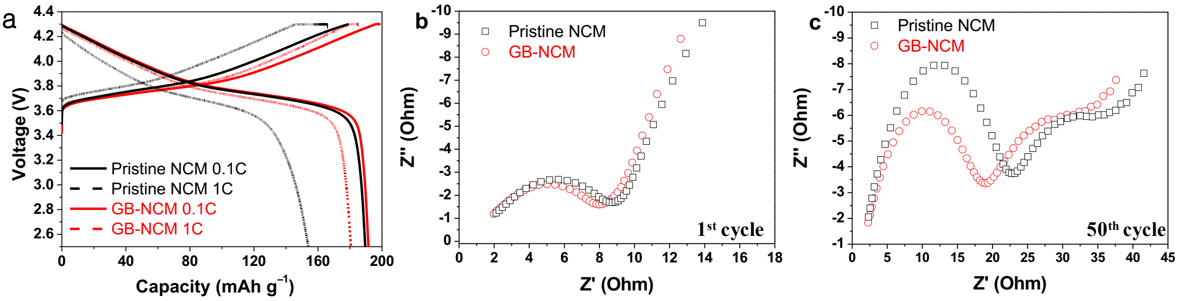

Supplementary Figure 7 | The electrochemical properties of both NCM electrodes at 25 °C. a, 1st charge-discharge profiles at 0.1C and 1C. **(b-c)** Electrochemical impedance spectroscopy (EIS) plots after **b,** 1st cycle and **c,** 50th cycle.

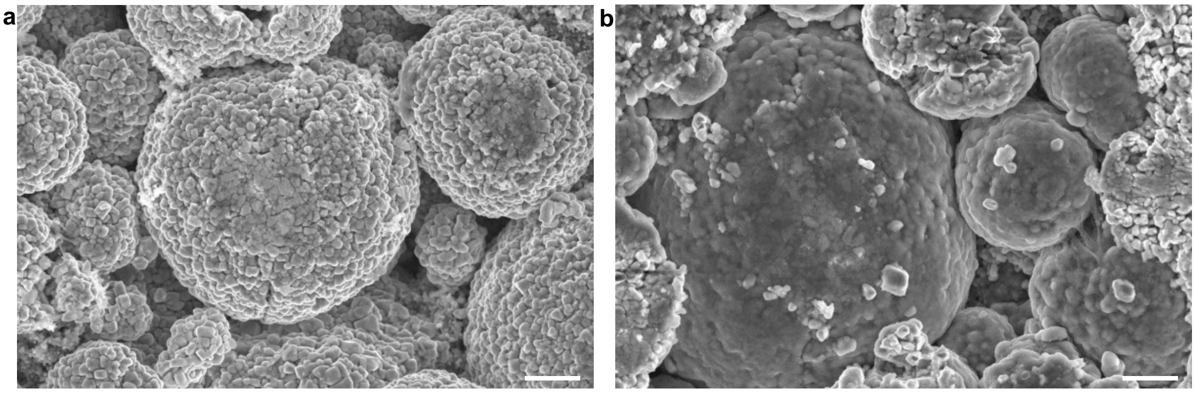

Supplementary Figure 8 | SEM images after 100 cycles. Battery measurement condition: half-cell, 60 ^{o}C, top cut-off =4.3V. **a,** pristine NCM (*scale bar*, 2 μm). **b,** GB-NCM (*scale bar*, 2 μm).

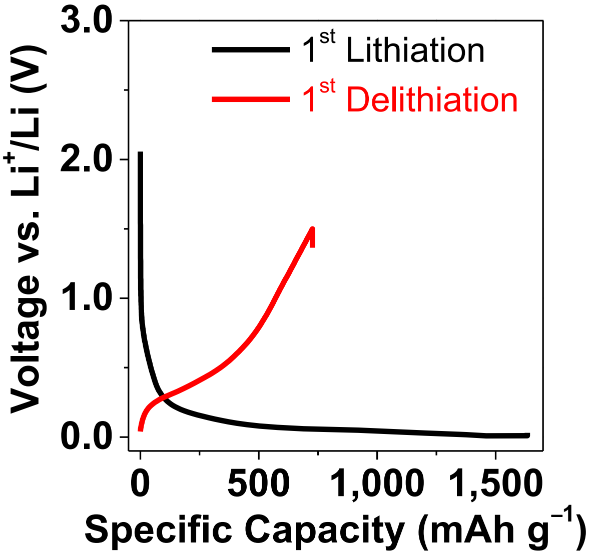

Supplementary Figure 9 | The 1st charge-discharge profiles of the GB anode at 0.1C and 25 °C. Areal capacity=2.7 mAh cm^{-2}.

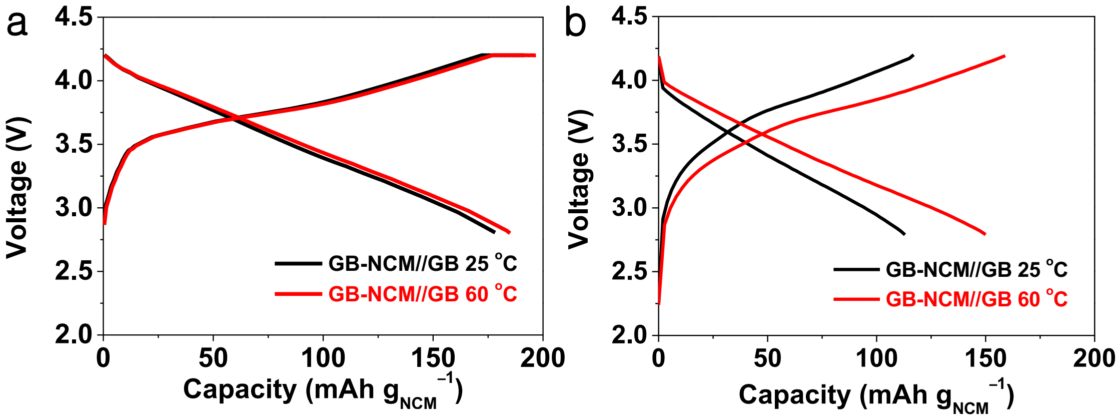

Supplementary Figure 10 | Charge-discharge profiles of the GB-NCM//GB full-cell. a, The first cycle at 0.1C. **b,** The second cycle at 25 °C and 60 °C when measured at 5C.

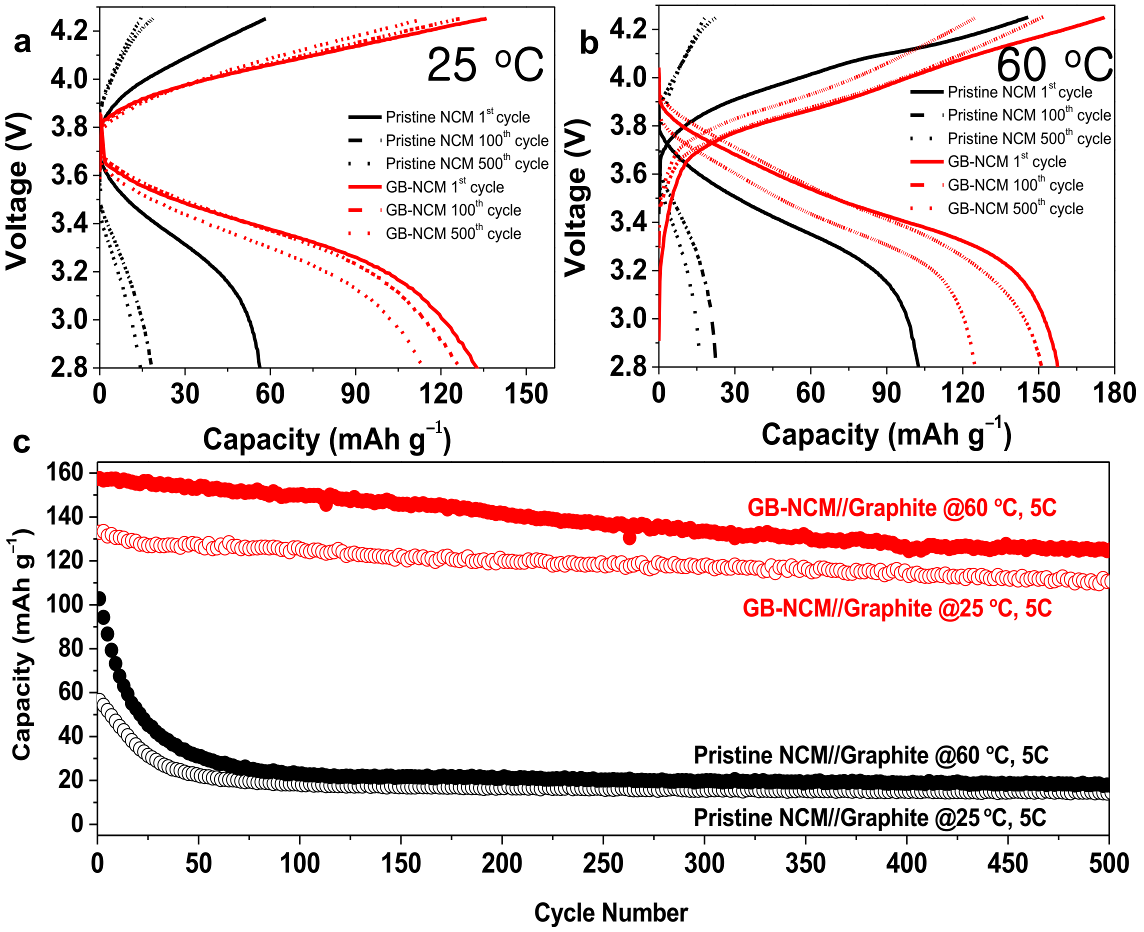

Supplementary Figure 11| The cycling performance and fast charging capability of NCM//graphite full-cells. (a-b) Charge-discharge profiles at **a,** 25 °C and **b,** 60 °C when measured at 5C (1C=190 mAh g_{NCM}^{-1}). **c,** Capacity retentions at 5C. The areal capacities of all the cells in this figure are 1 mAh cm^{-2}. In each cycle, charging and discharging rates were the same for all the data shown in this figure. Note that once the areal capacity is increased to 2.4 mAh cm^{-2} as in Figure 5 in the main text, the graphite anode does not operate as well as GB, because the graphite anode cannot afford to accommodate Li ions at this high C-rate during charge.

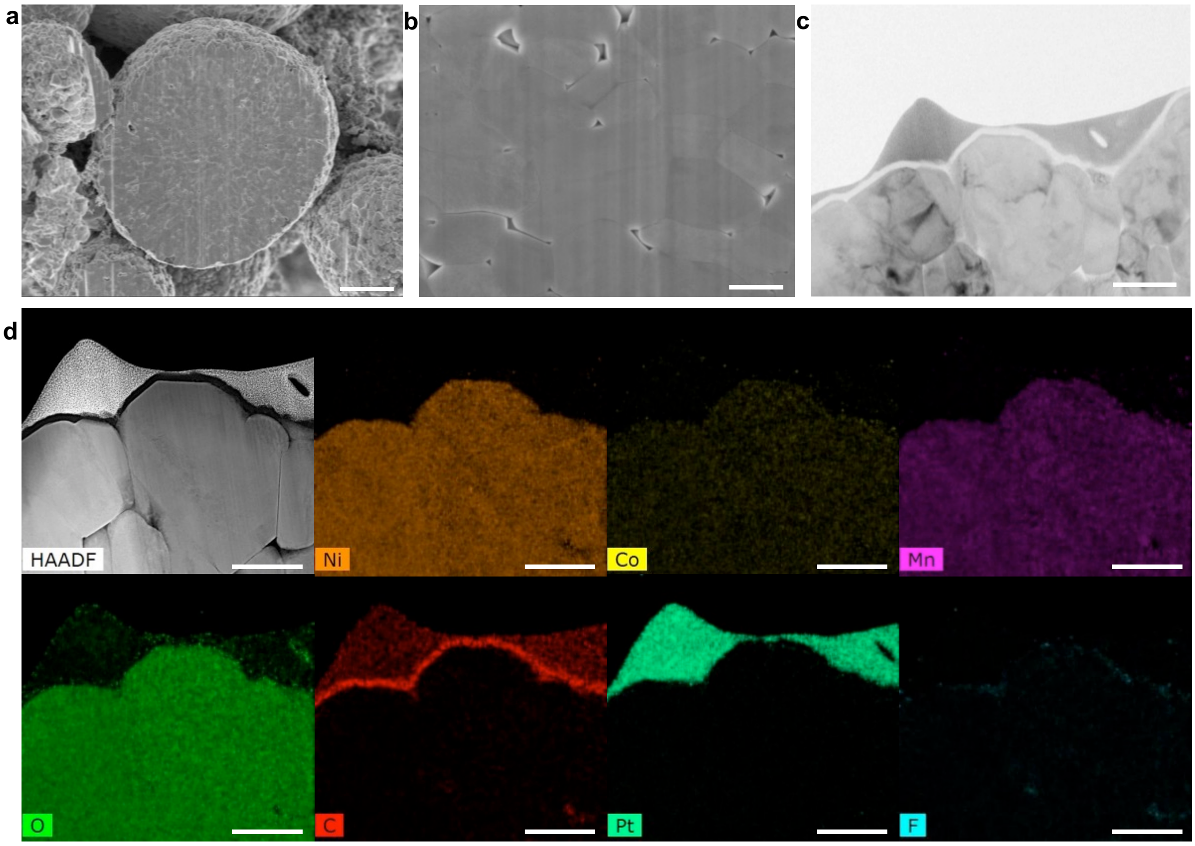

Supplementary Figure 12 | Characterization of the cross-section of GB-NCM after 500 cycles in full-cell at 5C and 60 °C. a-b, Cross-sectional SEM images (*scale bars*, 2 μm and 200 nm, respectively). **c,** STEM image (*scale bar*, 200 nm)**. d,** STEM image and its EDX elemental mapping with respect to Ni, Co, Mn, O, C, Pt, and F (*scale bars*, 200 nm).

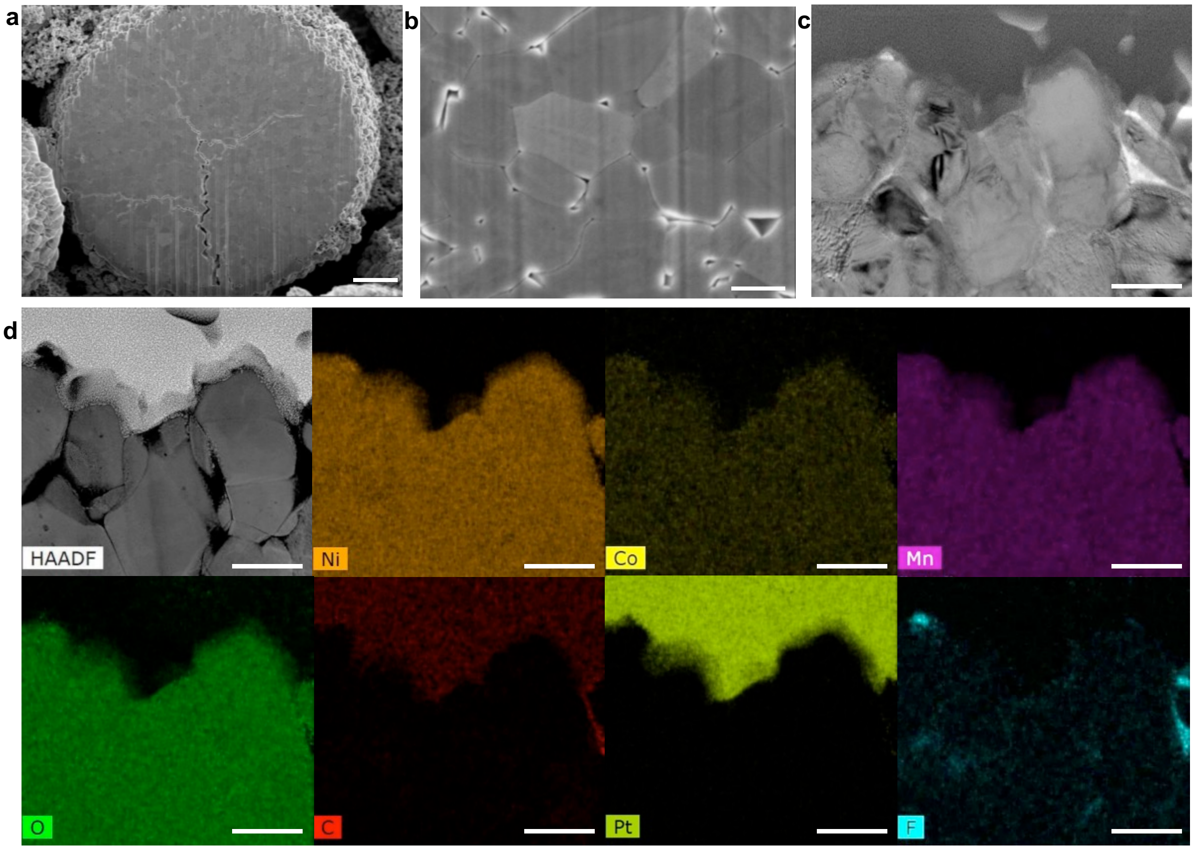

Supplementary Figure 13 | Characterization of the cross-section of pristine NCM after 200 cycles in full-cell at 1C and 25 °C. a-b, Cross-sectional SEM images (*scale bars*, 2 μm and 200 nm, respectively). **c,** STEM image (*scale bar*, 200 nm)**. d,** STEM image and its EDX elemental mapping with respect to Ni, Co, Mn, O, C, Pt, and F (*scale bars*, 200 nm). The given full-cell was cycled in a milder cycling condition in terms of temperature, C-rate, and cycle number, compared with the GB-NCM shown in Supplementary Fig. 11 because the capacity fading is too significant to reach the same number of cycles. The capacity fading is attributed to amplified transition metal dissolution that impairs the interfacial stability of the graphite anode.

Supplementary Tables

Supplementary Table 1 | Design parameters for an EV prismatic cell and specific capacities and energy densities using pristine NCM//graphite and GB-NCM//GB.

	Parameter	NCM//graphite	GB-NCM//GB
EV Cell	Current density (mAh cm^{-2})	4.4	4.4
	NP ratio	1.08[a]	1.0[a]
	Packing ratio (%)	90.0	90.0
	Energy Density (Wh L^{-1})	630	800
	Total capacity (Ah@0.33C)	**49.67**	**63.40 (Δ27.6%↑)**
Negative electrode	Capacity (mAh g^{-1})	360	716
	1st Efficiency (%)	95.0	99.0
	Electrode density (g cc^{-1})	1.6	1.1
	Content of active material (%)	97.5	95
Positive electrode	Capacity (mAh g^{-1})	190	190
	1st Efficiency (%)	93	93
	Electrode density (g cc^{-1})	3.0	4.0
	Content of active material (%)	92	97

[a]The different NP ratios originate from different initial Coulombic efficiencies (ICEs) of electrode materials. In the NCM//graphite cell, the ICEs of the NCM and graphite are ~93% and ~95%, respectively. With these ICE values, NP ratio of around 1.1 is widely adopted to have an excessive capacity in the anode. In the GB-NCM//GB cell, pre-lithiated GB has a higher ICE of 99% so that NP ratio was decreased to 1.0.

Supplementary Table 2 | Material-based energy densities obtained from ($C_{cathode}\cdot C_{anode}\cdot V_{nominal}$)/ ($C_{cathode} + C_{anode}$)

Cell type	C_{anode}		$C_{cathode}$		$V_{nominal}$	Energy density	
	[mAh g^{-1}]	[mAh cc^{-1}]	[mAh g^{-1}]	[mAh cc^{-1}]	V	[Wh kg^{-1}]	[Wh L^{-1}]
pristine NCM//graphite	360	576	190	570	3.4	422.8	974.1
GB-NCM//GB	700	770	190	760	3.4	508.1 (Δ20.2%↑)	1300.4 (Δ33.5%↑)

Supplementary Table 3 | Summary of electrochemical properties of $LiNi_{0.6}Co_{0.1}Mn_{0.3}O_2$ (NCM) in half-cell measurements at 25 °C and 60 °C.

25 °C		Capacity @ 0.1C (mAh g_{NCM}^{-1})	initial Coulombic efficiency (%)	Average Coulombic efficiency for 2-100 cycles (%)	Capacity retention for 2-100 cycles (%)	
Cut off 4.3 V	Pristine NCM	189.5	93.15	99.92	97.8	
	GB-NCM	192.2	93.71	99.96	99.5	Δ1.7%↑
Cut off 4.4 V	Pristine NCM	198.5	92.06	99.78	95.0	
	GB-NCM	201.1	92.32	99.93	98.5	Δ3.5%↑
Cut off 4.5 V	Pristine NCM	210.3	91.95	99.63	92.1	
	GB-NCM	211.5	92.89	99.90	97.3	Δ5.2%↑

60 °C		Capacity @ 0.1C (mAh g_{NCM}^{-1})	initial Coulombic efficiency (%)	Average Coulombic efficiency for 2-50 cycles (%)	Capacity retention for 2-50 cycles (%)	
Cut off 4.3 V	Pristine NCM	184.5	96.10	99.64	93.22	
	GB-NCM	191.4	96.62	99.68	97.10	Δ4.16↑
Cut off 4.4 V	Pristine NCM	198.9	91.92	99.58	92.70	
	GB-NCM	206.5	95.71	99.62	95.07	Δ2.56↑
Cut off 4.5 V	Pristine NCM	209.5	89.54	99.33	79.91	
	GB-NCM	215.1	91.96	99.46	90.99	Δ13.9↑

Supplementary Table 4 | Metal ion dissolution tests for pristine NCM and GB-NCM using inductively coupled plasma-atomic emission spectroscopy. 0.1 g of the active materials were immersed in 20 mL of the electrolyte co-solvents (EC:EMC:DMC=3:4:3=*v*:*v*:*v*) at 50 °C for 1 h, 3 h, and 7 h.

Sample	Time (h)	Extract concentration (mg L^{-1})			
		Li	Mn	Co	Ni
Pristine-NCM	1	0.2	0.2	0.0	0.5
	3	0.4	0.6	0.2	1.2
	7	0.4	0.7	0.2	1.6
GB-NCM	1	0.1	0.0	0.0	0.0
	3	0.1	0.0	0.0	0.0
	7	0.2	0.0	0.0	0.0

Supplementary Table 5 | Summary of electrochemical properties of the GB anode in half-cell measurements at 25 ^{o}C and 60 ^{o}C.

Sample	Capacity @ 0.1C (mAh g_{GB}^{-1})	initial Coulombic efficiency @0.1C (%)	Average Coulombic efficiency for 2-500 cycles at 5C (%)	Capacity retention for 500 cycles at 5C (%)
25 ^{o}C	716.2	44.5	99.98	88.0
60 ^{o}C	718.4	41.5	99.97	86.1

Supplementary Table 6 | Summary of electrochemical properties in GB-NCM//GB full-cell measurements at 25 ^{o}C and 60 ^{o}C.

Sample	Capacity @0.1C (mAh g_{NCM}^{-1})	initial Coulombic efficiency @0.1C (%)	Average Coulombic efficiency for 2-500 cycles at 5C (%)	Capacity retention for 500 cycles at 5C (%)
25 ^{o}C	178.2	93.1	99.97	84.1
60 ^{o}C	185.0	94.0	99.95	78.6
